# Poly(2-(dimethylamino)ethyl methacrylate)-Grafted Amphiphilic Block Copolymer Micelles Co-Loaded with Quercetin and DNA

**DOI:** 10.3390/molecules29112540

**Published:** 2024-05-28

**Authors:** Radostina Kalinova, Pavel Videv, Svetla Petrova, Jordan Doumanov, Ivaylo Dimitrov

**Affiliations:** 1 Institute of Polymers, Bulgarian Academy of Sciences, Akad. G. Bonchev St., Bl. 103-A, 1113 Sofia, Bulgaria; 2 Department of Biochemistry, Faculty of Biology, Sofia University “St. Kliment Ohridski”, 8 Dragan Tzankov Blvd., 1164 Sofia, Bulgaria; pvidev@biofac.uni-sofia.bg (P.V.); spetrova@biofac.uni-sofia.bg (S.P.); doumanov@biofac.uni-sofia.bg (J.D.)

**Keywords:** multifunctional, graft copolymer, click, micelleplex, quercetin, DNA, codelivery, metabolic activity

## Abstract

The synergistic effect of drug and gene delivery is expected to significantly improve cancer therapy. However, it is still challenging to design suitable nanocarriers that are able to load simultaneously anticancer drugs and nucleic acids due to their different physico-chemical properties. In the present work, an amphiphilic block copolymer comprising a biocompatible poly(ethylene glycol) (PEG) block and a multi-alkyne-functional biodegradable polycarbonate (PC) block was modified with a number of poly(2-(dimethylamino)ethyl methacrylate) (PDMAEMA) side chains applying the highly efficient azide–alkyne “click” chemistry reaction. The resulting cationic amphiphilic copolymer with block and graft architecture (MPEG-*b*-(PC-*g*-PDMAEMA)) self-associated in aqueous media into nanosized micelles which were loaded with the antioxidant, anti-inflammatory, and anticancer drug quercetin. The drug-loaded nanoparticles were further used to form micelleplexes in aqueous media through electrostatic interactions with DNA. The obtained nanoaggregates—empty and drug-loaded micelles as well as the micelleplexes intended for simultaneous DNA and drug codelivery—were physico-chemically characterized. Additionally, initial in vitro evaluations were performed, indicating the potential application of the novel polymer nanocarriers as drug delivery systems.

## 1. Introduction

Natural and synthetic polymers are promising platforms for the design of a great variety of delivery systems for biologically active (macro)molecules [1,2]. The progress in the development of controlled polymer synthesis and modification techniques has opened the possibility for the precise design and preparation of biocompatible and biodegradable polymer-based nanocarriers with finely tuned composition, functionality, and properties intended for different targeted therapies [3,4,5]. Thus, depending on composition and surface functionality, the drug carriers are capable of reaching the specific site of interest and delivering their cargo via the enhanced permeation and retention (EPR) effect or by active targeting [6,7,8]. Among the different types of polymer-based nanosystems, polymer micelles with a core-shell structure formed through self-association in the aqueous media of amphiphilic copolymers have demonstrated great potential as drug delivery systems [9,10,11]. Due to the fact that most of the drugs applied in cancer therapy are lipophilic, they can be accommodated into the micelles’ hydrophobic core for safe systemic circulation and delivery to the target tissue. Double hydrophilic copolymers comprising positively charged and neutral water-soluble segments, on the other hand, are extensively used in gene therapy to condense via electrostatic interactions and thus protect various biomacromolecules such as RNA or DNA [12]. The formed stable nanosized polyplexes should be able to prevent enzymatic degradation via nucleases in the blood and destabilization through electrostatic interactions with serum proteins during systemic circulation [13]. Further development of non-viral, polymer-based gene delivery systems involves the use of polymer micelles with positively charged surfaces for nucleic acid condensation. Thus, the building blocks for complex formation are nanosized particles, not individual polymer chains and the micellar gene delivery systems are referred to as micelleplexes [14,15]. The amphiphilic copolymers used for micelleplex formation comprise a biodegradable hydrophobic block forming the micelles’ core, while the hydrophilic shell-forming blocks are usually polycations such as polyethyleneimine (PEI), poly(2-(dimethylamino)ethyl methacrylate) (PDMAEMA), and poly(L-lysine) (PLL) [16]. Additionally, the charged hydrophilic shell can be mixed with neutral biocompatible polymer chains in order to improve stability and reduce the toxicity characteristic of the high molar-mass polycations [17]. The formation and evaluation of micelleplexes between genetic material and various amphiphilic cationic copolymers of linear, graft, and star architecture were reported [18,19,20,21]. It was established that the densely grafted (bottlebrush) cationic amphiphilic copolymers outperform their linear polycation analogs as pDNA nanocarriers in terms of enhanced gene expression and lower toxicity [22]. Another advantage of the micelleplex strategy is the possibility of constructing highly efficient drug carriers for the simultaneous delivery of more than one therapeutic agent to the target tumor cells [23,24]. In addition to the nucleic acid condensation, the core of the cationic micelles is capable of accommodating hydrophobic drugs. The potential synergistic effect of dual drug and gene delivery is expected to enhance the antitumor efficiency, overcoming the acquired multidrug resistance (MDR), which is a common phenomenon encountered in cancer therapy [25,26]. Thus, micelleplexes were evaluated as gene and drug codelivery systems in several reports. These include the micelleplexes formed from DNA and cationic amphiphilic copolymers containing encapsulated hydrophobic drugs such as doxorubicin (DOX) and camptothecin (CPT) [27,28]. Small interfering RNA (siRNA)-containing micelleplexes were also evaluated for the codelivery of paclitaxel (PTX), amphotericin B (AmB), cisplatin prodrug, and DOX [29,30,31,32]. siRNA-based micelleplexes were also used for the codelivery of 7-ethyl-10-hydroxycamptothecin (SN-38) and ultra-small superparamagnetic iron oxide nanoparticles for theranostic application [33].

Quercetin (Que, 2-(3,4-dihydroxyphenyl)-3,5,7-trihydroxy-4H-chromen-4-one) is a widely distributed natural flavonoid. It is characterized by anticancer, antioxidant, antimicrobial, antidiabetic, and anti-inflammatory activity [34]. Other pharmacological properties of quercetin such as antiviral, antihypertensive, and hepatoprotective effects have also been reported [35]. However, Que has a poor water solubility and a short half-life span in body fluids resulting in low bioavailability, that limits its therapeutic use. In order to overcome these limitations Que has been encapsulated into various nanocarriers including polymer micelles [36]. The codelivery of Que with antigen, proteins, or RNA using emulsions [37], liposomes [38,39], niosomes [40], lipid [41], and lipid-copolymer [42] nanoparticles has also been described. To the best of our knowledge, there are no reports in the literature concerning the development of micelleplexes intended for the codelivery of DNA and Que.

Herein, we present the synthesis of a novel amphiphilic copolymer comprising a biocompatible polyoxyethylene block and a hydrophobic biodegradable polycarbonate block grafted with PDMAEMA-side chains intended for DNA and Que codelivery. Initially, well-defined α,ω-heterobifunctional PDMAEMA was synthesized via atom transfer radical polymerization (ATRP). In the next step, a previously synthesized multifunctional amphiphilic diblock copolymer [43] was used as a modular platform for the PDMAEMA grafts attachment by applying the highly efficient azide–alkyne “click” reaction. The copolymer is self-associated in aqueous media with the formation of colloidally stable nanosized micelles. The micelles were loaded with Que followed by micelleplex formation through electrostatic interactions with DNA. Both types of nanosized aggregates (micelles and micelleplexes) were physico-chemically characterized. Furthermore, some initial in vitro evaluations were performed in order to assess the particles’ potential as drug and DNA codelivery nanovehicles.

## 2. Results and Discussion

### 2.1. Synthesis of Cationic Amphiphilic Graft Copolymer MPEG-b-(PC-g-PDMAEMA)

**Scheme 1:**
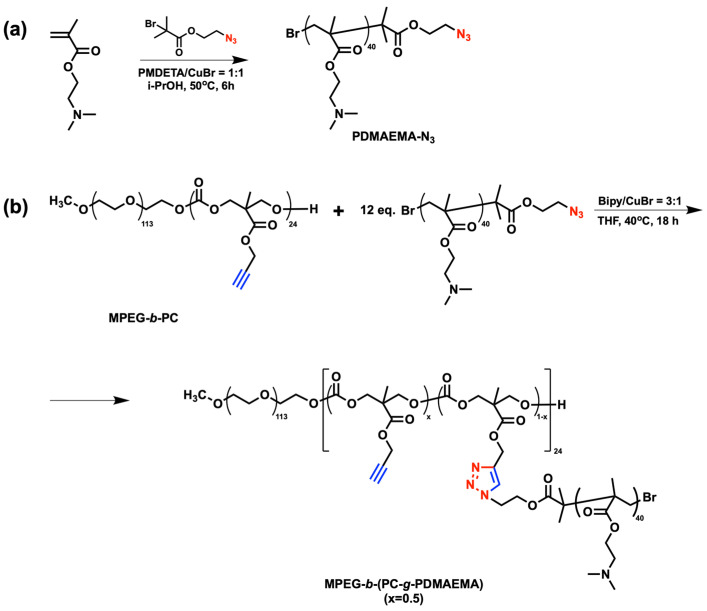
Synthetic path towards (**a**) azido-end functional PDMAEMA; (**b**) cationic amphiphilic graft copolymer MPEG-*b*-(PC-*g*-PDMAEMA).

We have recently reported the green synthesis and characterization of an amphiphilic block copolymer comprising a biocompatible polyoxyethylene block and a biodegradable polycarbonate block bearing alkyne side group functionality on each repeating unit (MPEG-*b*-PC). The block copolymer obtained was capable of encapsulating various hydrophobic biologically active compounds into the core of the formed micelles in aqueous media [43,44]. Furthermore, MPEG-*b*-PC was converted into a highly amino-functional double hydrophilic block copolymer and used for the formation of polyion complex micelles and polyplexes through electrostatic interactions with oppositely charged synthetic block copolymer or DNA [45]. Herein, we extend the possibilities of using the MPEG-*b*-PC copolymer as a modular platform for the preparation of cationic amphiphilic copolymer with a block and graft architecture. Initially, we synthesized well-defined azide-end functional PDMAEMA under ATRP conditions using a commercially available bifunctional initiator azido-Bib (Scheme 1a).

**Figure 1:**
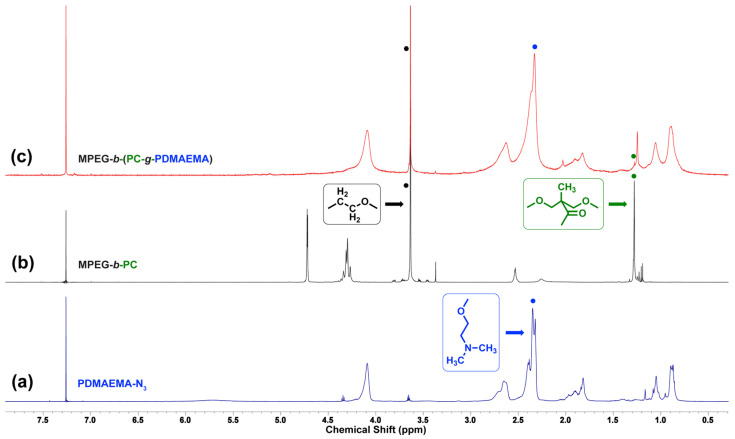
^1^H NMR spectra in CDCl_3_ of (**a**) azide end-functional polymer (PDMAEMA-N_3_); (**b**) alkyne-multifunctional amphiphilic block copolymer backbone (MPEG-*b*-PC); and (**c**) cationic amphiphilic graft copolymer MPEG-*b*-(PC-*g*-PDMAEMA).

The polymerization was performed in 2-propanol and was completed in 6 h, yielding an α,ω-bifunctional PDMAEMA. The degree of polymerization was calculated from the product’s ^1^H NMR spectrum recorded after the purification step (Figure 1a). The average degree of the DMAEMA polymerization of 40 was estimated from the relative intensities of methylene protons assigned to the monomeric repeating units at 4.09 ppm and those corresponding to the azide functional initiator at 4.34 ppm. The obtained value is somewhat higher than the theoretical one (t-*DP*_n_ = 30), which might be attributed to the low-level occurrence of side reactions originating from the azide end-groups. The same observations have been reported by others when using similar unprotected azide-containing ATRP initiators for the synthesis of PDMAEMA [46].

Nevertheless, the polymer obtained is well-defined and characterized by monomodal molar-mass distribution and a relatively low dispersity value of 1.27 (Figure S1, Table 1). The use of a functional initiator ensures the presence of an azide group on each polymer chain end. The azide functionality was also confirmed from the FTIR spectrum of the polymer in which the corresponding band at 2102 cm^−1^ is clearly visible (Figure S2a).

In the next step, the functional PDMAEMA was used for the synthesis of the cationic amphiphilic copolymer with a complex architecture. A “grafting to” approach was applied to attach the preformed PDMAEMA-N_3_ chains to the multi-alkyne functional amphiphilic backbone MPEG-*b*-PC (Scheme 1b). The highly efficient azide–alkyne “click” reaction was applied to graft the targeted average number of PDMAEMA side chains. The reaction was performed in THF using bipyridyl/CuBr in a molar ratio of 3:1 as a catalytic system. The final product was thoroughly purified and subjected to analyses. The azide band at 2102 cm^−1^ completely disappeared from the FTIR spectrum of the purified graft copolymer, indicating that there are no unreacted PDMAEMA-N_3_ chains left (Figure S2b). Moreover, the graft copolymer is characterized by a low dispersity as estimated from the GPC analysis (Table 1). Furthermore, there is a clear shift in the GPC trace towards higher molar mass compared to the backbone copolymer after the grafting reaction (Figure S1). The most intensive signals in the backbone polymer ^1^H NMR spectrum are those characteristic of the oxymethylene protons from the polyether chain at 3.64 ppm (Figure 1b). They are clearly visible in the ^1^H NMR spectrum of the grafted product together with the highly intensive signals for the methylene and dimethylamine protons of the PDMAEMA side chains at 4.09 and 2.33 ppm, respectively (Figure 1c). Knowing the molar masses of both the backbone polymer (MPEG-*b*-PC) and the single-side chain, the average degree of PDMAEMA grafting was estimated from the ratio between the integral intensities of protons at 3.64 ppm and those at 2.33 ppm. The obtained value corresponds to 50% grafting degree or approx. 12 PDMAEMA side chains per 1 backbone, which is close to the theoretical one. The molar mass characteristics of the precursors and the final graft copolymer are summarized in Table 1.

### 2.2. Self-Association of MPEG-b-(PC-g-PDMAEMA) Amphiphilic Graft Copolymer

The successfully synthesized graft copolymer possesses hydrophilic biocompatible nonionic polyether block and polycationic PMDAEMA grafts attached to a hydrophobic biodegradable polycarbonate backbone. The presence of hydrophobic and hydrophilic segments in the obtained macromolecular architecture could lead to the formation of self-assembled nanostructures in an aqueous medium. In order to check this assumption a nanoprecipitation technique for micelles formation was applied. Initially, a predetermined amount of graft copolymer was dissolved in the common for all constituting segments solvent acetone, which is also miscible with water. The organic solution was then added dropwise to stirred water. Following the acetone removal, the copolymer concentration in the aqueous phase was adjusted to the desired concentration and subjected to analyses.

**Figure 2:**
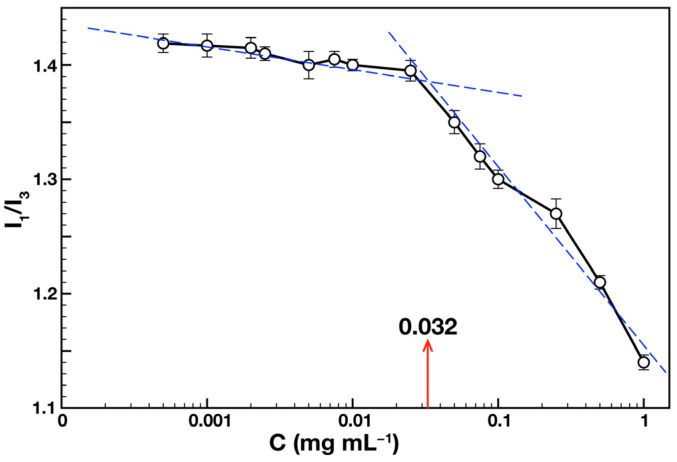
CMC determination for MPEG-*b*-(PC-*g*-PDMAEMA) graft copolymer: I_1_/I_3_ ratios from pyrene excitation spectra vs. copolymer concentration.

One of the most important parameters that gives an insight into the nanoaggregates’ thermodynamic stability is the critical micelle concentration (CMC). CMC is defined as the lowest concentration above which micelles start to form spontaneously. In the current study, the CMC of the newly obtained MPEG-*b*-(PC-*g*-PDMAEMA) graft copolymer was determined via fluorescence spectroscopy. The aqueous graft copolymer solutions were prepared at various concentrations in the presence of pyrene as a fluorescent probe. The CMC value was obtained from the plot of pyrene 1:3 relative intensity (I_1_/I_3_) versus copolymer concentration. Below CMC the I_1_/I_3_ value corresponds to a polar environment, whereas when the micelles start to form the I_1_/I_3_ ratio decreases rapidly, indicating that pyrene is sensing the hydrophobic environment of micelles’ core. The CMC value of 0.032 mg mL^−1^ for the graft copolymer was extracted from the plot presented in Figure 2.

The obtained low value is in the micromolar range (0.37 μM) and indicates that the formed aggregates would be stable upon plasma dilution for potential drug delivery applications. The following DLS measurements of the graft copolymer particles were performed at a concentration of 1 mg mL^−1^, which is far above the determined CMC.

**Figure 3:**
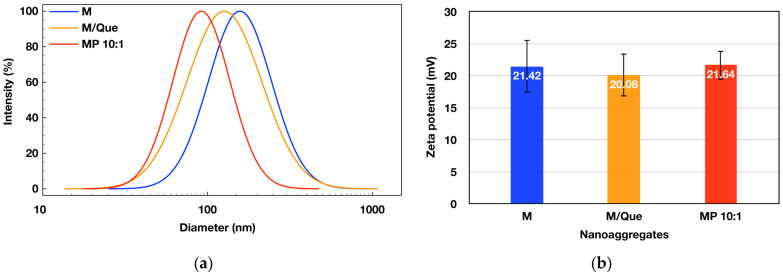
Size distributions (**a**) and zeta potentials (**b**) obtained from dynamic light scattering measurements of aqueous dispersions of empty micelles (M: d = 157.91 ± 2.42 nm, PdI: 0.221, ζ = 20.08 ± 3.73 mV), quercetin-loaded micelles (M/Que: d = 126.88 ± 2.26 nm, PdI: 0.312, ζ = 21.42 ± 4.10 mV), and quercetin-loaded micelleplexes prepared at N/P = 10:1 (MP 10:1: d = 91.80 ± 1.49 nm, PdI: 0.173, ζ = 21.64 ± 1.65 mV).

The DLS measurements confirmed the presence of nanosized copolymer aggregates in aqueous media (Figure 3). The micelles are characterized by average diameters of around 160 nm and a relatively narrow size distribution (Figure 3a, M). The strongly positive surface charge expressed by the measured particles’ zeta potential of 21 mV is due to the micelles’ shell formed by the PDMAEMA grafts (Figure 3b, M). The positive surface of the micelles contributes to their enhanced colloidal stability.

**Figure 4:**
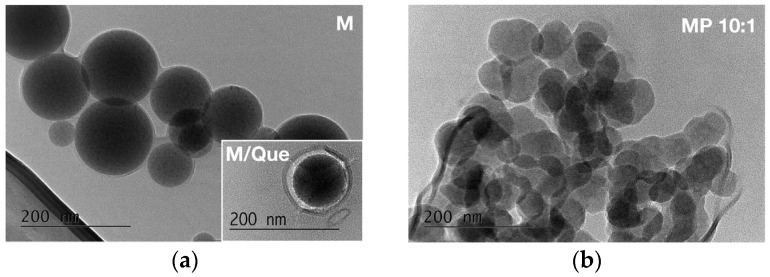
TEM micrographs of (**a**) graft copolymer micelles (M). The inset shows the quercetin-loaded micelle (M/Que) and (**b**) quercetin-loaded micelleplexes obtained at N/P = 10:1 (MP 10:1).

The graft copolymer micelles were further studied using transition electron microscopy in order to visualize their morphology (Figure 4a). The TEM images reveal the spherical shape of the micelles. The measured average particles’ diameters are similar to those obtained from the DLS analyses. The slight differences in measured sizes detected using the two methods could be attributed to the different conditions under which the samples were evaluated via DLS and TEM analyses.

### 2.3. Drug-Loading and In Vitro Release Studies

The next step in the current study was to evaluate the possibility of hydrophobic drug loading into the core of the graft copolymer micelles. Quercetin was chosen as a model hydrophobic drug since it possesses numerous biological activities and, at the same time, requires protection during the systemic circulation toward the target cells. The hydrophobic drug was encapsulated into the copolymer micelles during the process of their formation. A common solution of the predetermined amounts of quercetin and the graft copolymer was prepared in acetone. The organic solution was added dropwise to stirred water, and the Que-loaded micelles were obtained similarly to the procedure used for the empty micelles. The concentration of the aqueous dispersion was adjusted to 1 mg mL^−1^ with a graft copolymer-to-drug ratio of 10:1 (wt/wt). As a final step, the aqueous micelles dispersion was passed through a 0.45 μm membrane filter in order to separate the unloaded drug from the micellar quercetin.

The ability of the cationic graft copolymer micelles to effectively encapsulate the hydrophobic active compound was evaluated through the drug loading efficiency (DLE) and drug loading capacity (DLC) parameters. In order to quantify the amount loaded into the micelles Que, the aqueous micellar dispersions were lyophilized, followed by the resuspension of a predetermined amount of dry material in ethanol. The solutions were subjected to UV analyses and the amount of the encapsulated drug was determined from the absorption intensity at λ_max_ = 374 nm. The high DLE = 93% and DLC = 8.5% for the novel copolymer micellar system were calculated by applying Equations (1) and (2) (see Section 3).

The quercetin-loaded micelles (M/Que) were also evaluated using DLS and TEM analyses. The DLS measurements reveal that the drug-loaded micelles exhibit somewhat smaller average diameters compared to those of the empty micelles (Figure 3a). The formation of more compact structures might be explained by the interactions occurring between the loaded Que and the polymer chains forming the core of the hydrophobic micelles. On the other hand, there is just a negligible change in the Que-loaded micelles’ zeta potential (Figure 3b). The similar surface charge detected for both empty and loaded micelles is an indication that most of the drug is located in the hydrophobic core or in its vicinity. The slightly smaller size of the Que-loaded micelles compared to the empty ones was also visualized in the TEM analysis, whereas the spherical shape of the loaded micelles is preserved (Figure 4a, inset).

**Figure 5:**
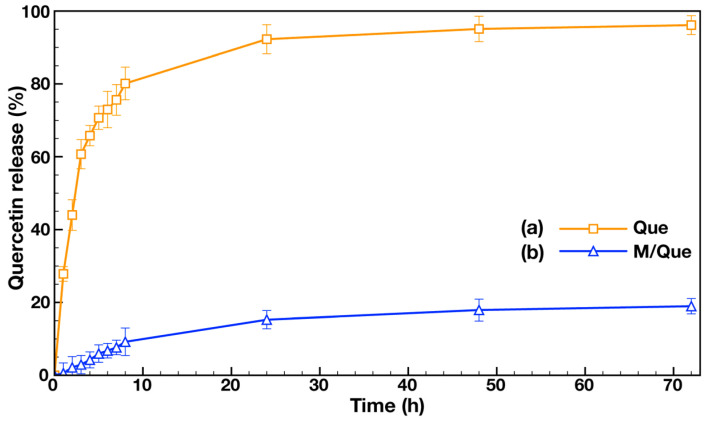
In vitro release profiles in phosphate buffer (PBS, pH 7.4) containing 10% (*v*/*v*) ethanol of (**a**) free quercetin (Que) and (**b**) quercetin from the cationic graft copolymer micelles (M/Que). The data are expressed as mean value ± SD, n = 3.

In vitro, dissolution tests were performed on the quercetin-loaded micelles in order to assess the drug release profile. The release media ensuring the sink conditions comprised phosphate buffer (pH 7.4) and ethanol in a 9:1 (*v*/*v*) ratio. The aqueous micellar dispersion (5 mL, 1 mg mL^−1^) diluted with an equal volume from the release media was introduced into a dialysis membrane bag (MWCO 50,000 Da). The bag was immersed into the stirred release media and the whole system was kept at 37 °C. Samples from the release media were withdrawn at specific time intervals and analyzed using UV/Vis spectroscopy. The sink conditions were maintained via the addition of fresh release media each time a sample was withdrawn. For comparison, the release profile of the free quercetin was also obtained under the same conditions. The cumulative quercetin release versus time profiles for the two systems are shown in Figure 5. The free drug shows a high release rate. During the first eight hours of the evaluation, approximately 80% of the drug has migrated through the membrane into the release media. At the 24th hour, more than 90% of Que was detected in the release media (Figure 5a). On the contrary, only a small amount of the drug was released from the cationic graft copolymer micelles. During the first eight hours of evaluation, just 9% of Que was released from the micelles, followed by a sustained drug release, reaching 19% after 72 h of evaluation. The initial faster rate could be attributed to the release of a small amount of Que that is located in the micelles’ shell in the vicinity of the hydrophobic core. The following very slow and sustained drug release is due to the release of Que from inside the core of the micelle. The reason for the slow and sustained Que release could be explained by the formation of hydrogen bonds and the hydrophobic interactions between the drug and micelles’ core-forming macromolecules [47]. This assumption is in agreement with the results from the DLS measurements, which indicated the formation of more compact nanostructures after the drug-loading step.

Overall, the results from the in vitro Que release evaluations performed at physiological pH conditions indicate that the active compound could be safely transported into graft copolymer micelles and sustainably released, thus preventing its clearance during systemic circulation.

### 2.4. Micelleplexes Formation between the Quercetin-Loaded Cationic Micelles (M/Que) and DNA

The final step in the study was to test the ability of the quercetin-loaded cationic micelles (M/Que) to condense DNA via electrostatic interactions. The micelleplexes were prepared by mixing equal volumes of aqueous DNA solution (100 μg mL^−1^) and Que-loaded micellar dispersions of various concentrations. Thus, different molar ratios between the graft copolymer cationic groups and the DNA phosphate groups (N/P) were achieved. After 1 h equilibration, the micelleplexes prepared at N/P = 1:1, 2.5:1, 5:1, 7.5:1, 10:1 and 20:1 were subjected to DLS measurements (Figure S3). The micelleplexes’ average diameters increased from 144 to 352 nm, with the N/P ratio increasing from 1:1 to 5:1. The further increase in the N/P ratio led to the formation of compact micelleplexes characterized by average diameters in the 80–112 nm range and narrow size distributions (Figure S3a). The zeta-potential measurements revealed that, except for the micelleplex prepared at N/P = 1:1 whose surface is highly negative (ζ = −19.2 mV), the rest of the particles exhibit positive surface charge with zeta potentials within the 19–24 mV range (Figure S3b). No significant changes in the zeta potential value were observed with the increase in the N/P ratio from 2.5:1 to 20:1. The results from DLS analyses indicate that the quercetin-loaded cationic micelles are capable of condensing DNA into sub-100 nm micellplexes at N/P ratio 10:1 and above.

**Figure 6:**
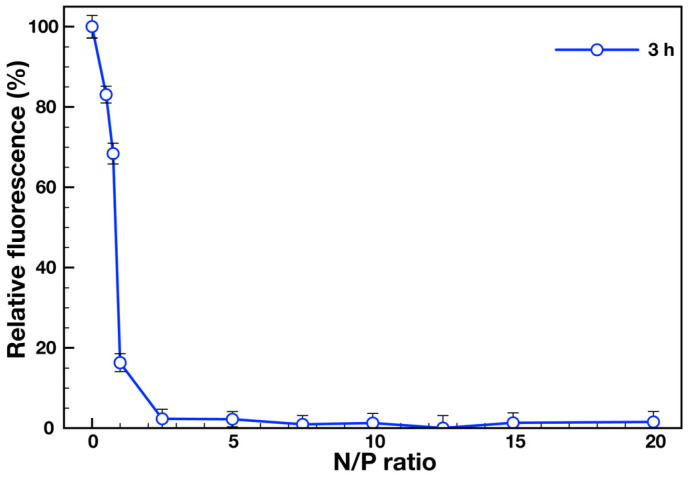
Ethidium bromide displacement assay for miclelleplexes prepared at different N/P ratios. The data are expressed as mean value ± SD, n = 3.

The binding ability of the cationic micelles to DNA was further evaluated by applying the ethidium bromide displacement assay. When intercalated into the DNA’s double helix, ethidium bromide leads to a significant enhancement of the nucleic acid’s fluorescent intensity. If such a complex is used for the preparation of micelleplexes, the binding ability of the micelles could be assessed by following the gradual quenching of the fluorescence intensity as a result of ethidium bromide displacement from its complex with DNA by the added micellar dispersion. The results from the fluorescence measurements of micelleplexes prepared at different N/P ratios using the EtBr–DNA complex are presented in Figure 6. It is clearly visible that, above an N/P ratio of 2.5, the fluorescent intensity is completely quenched. This is an indication of the strong binding ability of the cationic micelles and their potential to efficiently condense and preserve DNA during transport to the target cells.

Based on the results obtained, micelleplexes formed at an N/P ratio of 10:1 (MP 10:1) were chosen as the optimal formulation for further evaluation. They are characterized by an average diameter of 91.8 nm and narrow size distribution (PdI: 0.173) according to the DLS measurements (Figure 3a). The particles are positively charged with ζ = 21.6 mV (Figure 3b). The obtained TEM image reveals the presence of clusters of nanosized spherical micelleplexes with average diameters somewhat smaller but still close to those extracted from the DLS measurements (Figure 4b).

**Figure 7:**
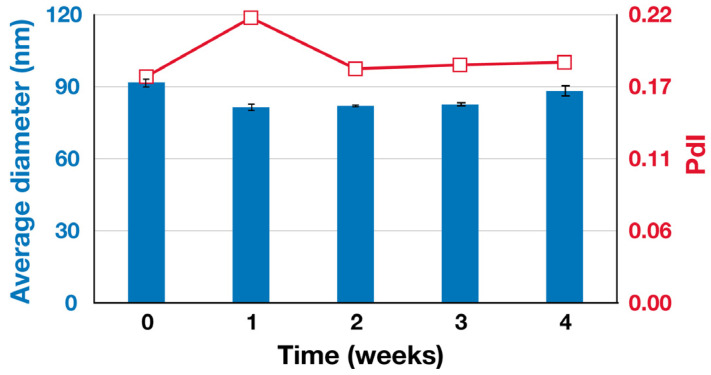
Stability of quercetin-loaded micelleplexes (MP 10:1) in aqueous media assessed using DLS measurements as a function of incubation time.

The Que-loaded micelleplexes’ colloidal stability was further evaluated as a function of time. The micelleplexes’ (MP 10:1) aqueous dispersions were incubated at room temperature and, at predetermined time intervals, the variations in their average diameters were followed by DLS measurements. The results showed that the drug-loaded micelleplexes remained stable after 4 weeks of incubation with negligible fluctuations in their sizes and size distributions (Figure 7). The demonstrated excellent stability of the drug-loaded micellar dispersions indicates that they might be suitable candidates for further evaluations as safe nanocarriers for drug and DNA codelivery.

Besides the micelleplexes’ stability over time, their capability to release the condensed DNA in acidic conditions characteristic of endosomes was also assessed. Thus, the aqueous micelleplex dispersion (MP 10:1) was incubated in an acidic buffer (pH 5.6) and the changes in particle sizes and size distribution were detected using DLS analyses. After an hour of the micelleplexes’ incubation in an acidic environment, significant changes in particle sizes and size distribution were observed. Different particle populations in the nano- and micrometer size range were detected (Figure S4). These were attributed to the micelles and the released DNA, respectively. These initial results show the potential capability of the micelleplexes to release the condensed DNA into the endosomes.

The main characteristics of the nanoaggregates obtained from the cationic amphiphilic graft copolymer MPEG-*b*-(PC-*g*-PDMAEMA) (empty or drug-loaded micelles and the optimal micelleplex formulation) are summarized in Table 2.

### 2.5. In Vitro Metabolic Activity Assessment of HepG2 Cells Treated with Empty or Quercetin-Loaded Micelles and Micelleplexes

**Figure 8:**
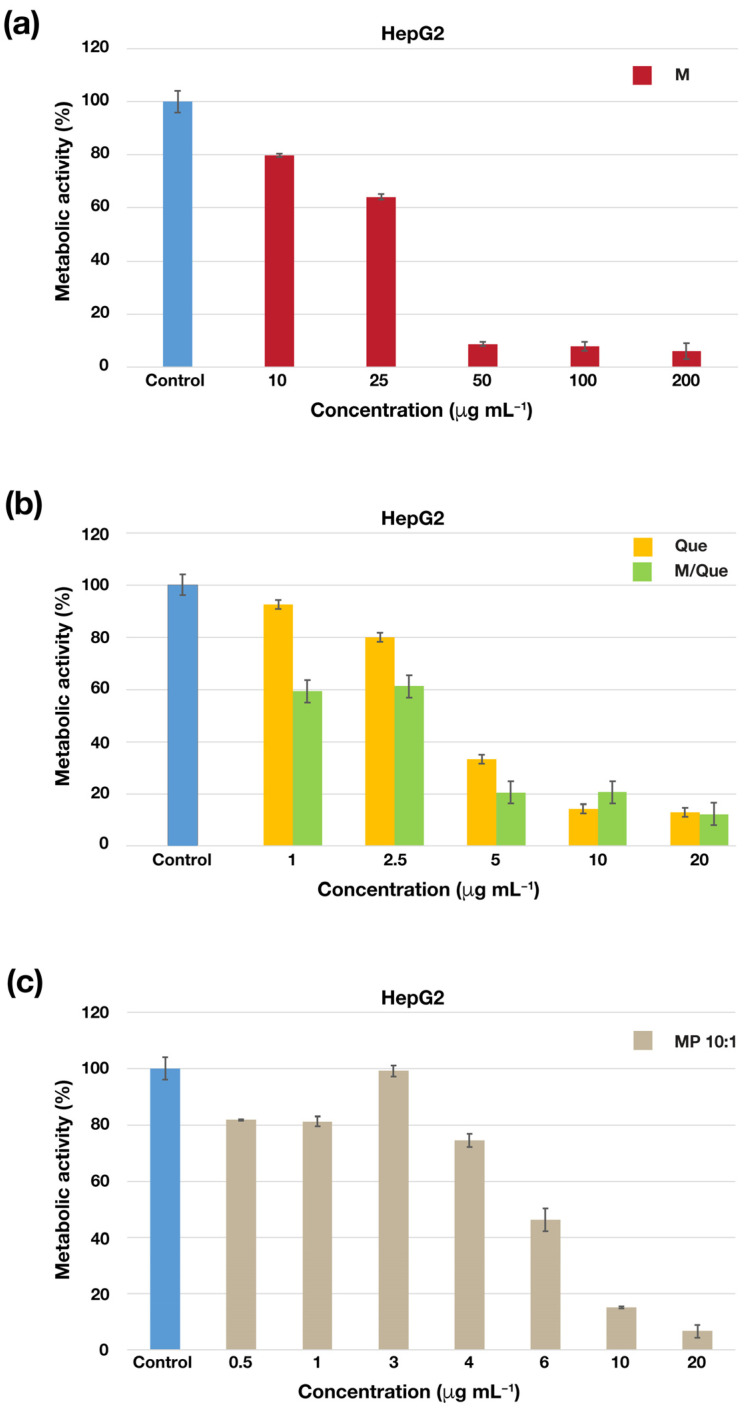
Cell metabolic activity defined via the MTT test after 24 h of incubation of HepG2 cells treated with different concentrations of (**a**) empty cationic graft copolymer micelles (M); (**b**) free (Que) and micellar (M/Que) quercetin; and (**c**) quercetin-loaded micelleplex obtained at N/P = 10:1 (MP 10:1). The data are presented as percentages of untreated controls and are expressed as mean value ± SE, n = 3.

The MTT test was performed using various types of nanoaggregates on the human liver cancer HepG2 cell line (Figure 8). Initially, the potential of the novel cationic graft copolymer micelles (M) as safe drug nanocarriers was evaluated. The concentration-dependent metabolic activity of HepG2 cells treated with the empty micellar dispersion in a wide concentration interval (10–200 μg mL^−1^) is presented in Figure 8a. The cationic micelles caused low cytotoxic effects on the cell line of up to concentrations of 25 μg mL^−1^ since they failed to induce 50% inhibition of metabolic activity. At higher micellar concentrations, the inhibition of cell metabolic activity became significant and was evaluated to be up to 94% compared to the untreated control cells. The significant cytotoxic effect of the empty micelles at higher concentrations can be explained by the presence of densely grafted PDMAEMA chains on the particles’ surface. However, this effect of the PDMAEMA graft architecture is much less pronounced compared to that of the linear PDMAEMA analogs [48]. The next step was to evaluate the in vitro metabolic activity of cancerous HepG2 cells treated with increasing concentrations of quercetin-loaded cationic micelles (M/Que), comparing it with the metabolic activity of cells treated with free quercetin (Que) used in the same concentrations and introduced as an ethanolic solution (Figure 8b). The results clearly demonstrated that in the concentration interval 1–5 μg mL^−1^, the micellar quercetin inhibits the metabolic activity of the cancer cell line to a greater extent (<30%) compared to the effect of the free drug. The concentration increase in both the free and micellar Que (5–20 μg mL^−1^) resulted in comparable values for metabolic activity. It might be concluded that the encapsulation of Que into the cationic graft copolymer micelles does not lead to a reduction in its cytotoxic effect on the cancerous cell line. Finally, in vitro evaluations on the metabolic activity of the HepG2 cell line treated with increasing concentrations of Que-loaded micelleplexes were carried out to evaluate the potential of the novel nanoparticles as safe carriers of both DNA and Que (Figure 8c). The results showed that the micelleplex (MP 10:1) used in the concentration range from 0.5 to 4 μg mL^−1^ caused only low cytotoxic effects on the cell line studied. The inhibition of cell metabolic activity was evaluated to be between 1 and 26% compared to the untreated control. At higher MP concentrations (6–20 μg mL^−1^), we observed above 50% inhibition of metabolic activity, most likely due to the increased content of PDMAEMA-grafts. Since the required DNA concentrations for the transfection protocols are between 1 and 2.5 μg mL^−1^ (where the studied micelleplexes show no cytotoxic effect), they can be considered as safe DNA and Que nanocarriers.

## 3. Materials and Methods

### 3.1. Materials and Reagents

All the reagents were purchased from Aldrich (Burlington, MA, USA) unless otherwise specified. 2-(Dimethylamino)ethyl methacrylate (DMAEMA, 98%) was purified by passing it through a column packed with Al_2_O_3_. Tehrahydrofuran (THF, ≥99.9%) was distilled from calcium hydride prior to use. A stock solution of 100 μg mL^−1^ salmon sperm DNA (*M*_w_ ~ 2000 bp) was prepared in ultra-pure water (>18 MΩ) and used for the polyplex formation. 2-Propanol (≥99.8%), ethanol (≥99.8%), acetone (≥99.9%), 2,2′-bipyridyl (bipy, ≥99%), 2-azidoethyl 2-bromoisobutyrate (Azido-Bib, ≥95%), *N,N,N′,N″,N″*-pentamethyldiethylenetriamine (PMDETA, 99%), copper(I) bromide (CuBr, 99.999%), pyrene (Py, ≥99%), ethidum bromide (EtBr, ~95%), and quercetin (Que, ≥ 95%) were used as received.

The multifunctional polyoxyethylene-*b*-polycarbonate amphiphilic block copolymer bearing alkyne pendant groups (MPEG-*b*-PC) was synthesized as previously described [43]. Briefly, the metal-free catalyzed ring-opening polymerization of an alkyne-functional cyclic carbonate monomer was initiated using a methoxy-poly(ethylene glycol) (MPEG) macroinitiator (*DP*_n (NMR)_ = 114) and was performed in bulk under green synthetic conditions. ^1^H NMR (600 MHz, CDCl_3_, δ, ppm): 4.72 (s, O-C*H*_2_-C≡CH), 4.30 (m, O-CH_2_-C*H*_2_-O(C=O) + O-C(O)-O-C*H*_2_)), 3.63 (s, O-*CH_2_*-*CH_2_*-O), 3.37 (s, C*H*_3_-O), 2.53 (s, CH_2_-C≡C*H*), and 1.28 (s, *CH_3_*). *DP*_n (NMR)_ = 24, *Ð*_M (GPC)_ = 1.24.

### 3.2. Synthesis of Azide End-Functionalized Poly(2-(dimethylamino)ethyl methacrylate) (PDMAEMA-N_3_)

The monomer (DMAEMA, 2 g, 2.14 mL, 12.72 mmol), azido-Bib (0.42 mmol, 0.1 g, 0.07 mL), PMDETA (0.07 g, 0.082 mL, 0.42 mmol), CuBr (0.42 mmol, 0.06 g), and 2.5 mL of 2-propanol were introduced into a Schlenk tube. The mixture was subjected to three freezing/pump-thawing cycles under a vacuum in order to remove the entrapped oxygen. The polymerization proceeded at 50 °C for 6 h in an argon atmosphere. The solvent was evaporated, and the residue was redissolved in THF and passed through a column containing neutral Al_2_O_3_. The solution was concentrated on a rotary evaporator and the polymer was recovered through precipitation into a cold hexane. Yield: 1.35 g (67.5%). ^1^H NMR (600 MHz, CDCl_3_, δ, ppm): 4.34 (t, O-C*H*_2_-CH_2_-N_3_), 4.09 (t, O-C*H*_2_-CH_2_-N), 3.65 (t, O-CH_2_-C*H*_2_-N_3_), 2.65 (t, CH_2_-C*H*_2_-N), 2.35 (s, N-(*CH*_3_)_2_), 2.05–1.65 (m, C*H*_2_-C(CH_3_)), 1.20–0.75 (m, CH_2_-C(C*H*_3_) + C-(C*H*_3_)_2_). *DP*_n (NMR)_ = 40, *Ð*_M (GPC)_ = 1.27, FTIR: 2102 cm^−1^.

### 3.3. Synthesis of Amphiphilic Polycationic Graft Copolymer MPEG-b-(PC-g-PDMAEMA)

MPEG-*b*-PC (0.05 g, 0.12 mmol alkyne groups), PDMAEMA-N_3_ (0.45 g, 0.072 mmol), and bpy (28.1 mg, 0.18 mmol) were placed in a round-bottom flask and dissolved in 5 mL of THF. The solution was degassed via argon bubbling for an hour. Then, CuBr (8.6 mg, 0.06 mmol) was added and the brown solution was stirred overnight under argon at 40 °C. The reaction mixture was diluted with THF and passed through a column packed with neutral Al_2_O_3_. The THF solution was concentrated on a rotary evaporator, diluted with water, and the polymer was further purified through ultrafiltration using a membrane with a molecular weight cut-off (MWCO) of 10,000 Da. The graft copolymer was recovered after lyophilization. Yield: 0.40 g, 80%. ^1^H NMR (600 MHz, CDCl_3_, δ, ppm): 7.78 (s, triazole); 5.27 (s, O-C*H*_2_-triazole), 4.72 (s, O-C*H*_2_-C≡CH), 4.50–4.00 (m, O-C*H*_2_-CH_2_-triazole + O-CH_2_-C*H*_2_-O(C=O) + O-C(O)-O-C*H*_2_) + O-C*H*_2_-CH_2_-N + O-CH_2_-C*H*_2_-triazole), 3.64 (s, O-*CH*_2_-*CH*_2_-O), 3.37 (s, C*H*_3_-O), 2.65–2.53 (t, CH_2_-C*H*_2_-N + s, CH_2_-C≡C*H*), 2.33 (s, N-(*CH*_3_)_2_), 2.10–1.75 (m, C*H*_2_-C(CH_3_)), 1.20–0.80 (m, CH_2_-C(C*H*_3_) + C-(C*H*_3_)_2_). Grafting degree: 50% _NMR_. *Ð*_M (GPC)_ = 1.22.

### 3.4. Preparation of Micelles

The polymeric micelles were formed through self-assembly of the PEG-*b*-(PC-*g*-PDMAEMA) copolymer as follows: first, the copolymer was dissolved in acetone, which is a good solvent for all three PEG, PC, and PDMAEMA segments, and the polymer concentration was adjusted to 20 mg mL^−1^. Then, the copolymer solution (2 mL) was added dropwise to ~16 mL ultrapure water under magnetic stirring (1000 rpm). The organic solvent (acetone) was removed on a rotary vacuum evaporator and the concentration of the micellar dispersion was adjusted to 1 mg mL^−1^ by the addition of ultrapure water.

### 3.5. Critical Micelle Concentration (CMC) Determination

The critical micelle concentration (CMC) of the newly synthesized cationic amphiphilic copolymer in aqueous media was determined using fluorescence spectrometry, using pyrene as the hydrophobic probe. Briefly, 10 µL from the 0.1 mM stock solution of pyrene in acetone were added to 1.0 mL of PEG-*b*-(PC-*g*-PDMAEMA) aqueous solutions with increasing polymer concentrations (from 5 × 10^−4^ to 1 mg mL). The final concentration of pyrene was 1 × 10^−6^ M. The solutions were kept at 25 °C and equilibrated for 24 h before fluorescent emission measurements with the excitation wavelength of 335 nm. The spectra were recorded at room temperature in the 355–600 nm wavelength range. The CMC was estimated from the ratio of pyrene intensities at 373 and 384 nm (I_1_/I_3_) versus the polymer concentration plot.

### 3.6. Drug-Loading Procedure

A similar protocol was used for the preparation of drug-loaded micelles: first, the Que solution in acetone at a concentration of 1 mg mL^−1^ was prepared. Then, the triblock copolymer (22 mg) was dissolved into 2.2 mL from the drug solution, and 2 mL from this solution were added dropwise to ~16 mL of ultrapure water under vigorous stirring. After the acetone removal via rotary vacuum evaporation, the concentration was adjusted to 1 mg mL^−1^, and the formed micellar dispersion was filtered (0.45 μm) to eliminate any unencapsulated quercetin. To evaluate the Que loading efficiency, the micellar dispersion was lyophilized, and the obtained dried sample was resuspended in ethanol. The solution was then analyzed using UV–Vis spectroscopy at a wavelength of 374 nm. A previously obtained value for the extinction coefficient, ε = 20,432 M^−1^ cm^−1^ (λ_max_ = 374 nm) of Que in ethanol, was used to quantify the amount of the encapsulated drug in the micelles [49]. The drug-loading efficiency (DLE) and drug-loading capacity (DLC) values were calculated based on the following equations:

DLE (wt%) = (the mass of encapsulated Que/the input mass of Que) × 100(1)

DLC (wt%) = (the mass of encapsulated Que/total mass of the micelles) × 100(2)

### 3.7. In Vitro Quercetin Release Profiles

A total of 5 mL from Que-loaded micelles dispersion (1 mg mL^−1^) diluted with an equal volume from the release media were placed into a dialysis membrane tubbing (MWCO 50,000 Da). The bag was immersed in 100 mL of release media comprising phosphate buffer (pH 7.4) and ethanol 9:1 (*v*/*v*). The system was kept at 37 °C under stirring (100 rpm). At predetermined time intervals 2 mL samples were withdrawn from the release media and the released amount of Que was detected using UV–Vis spectroscopy (λ_max_ = 379 nm). After each sample withdrawal, the same volume of fresh medium was added to keep the sink conditions. In an alternative experiment, the release profile of free Que was evaluated under the same conditions. The results were presented as a percentage of released drug vs. time. The release experiments were run in triplicate.

### 3.8. Micelleplexes Formation

The micelleplexes were formed by the dropwise addition of 2 mL from the salmon sperm DNA solution (100 μg mL^−1^) to equal volumes from the drug-loaded micellar dispersions prepared at different concentrations. The mixed dispersions were gently stirred at room temperature for 1 h. Thus, micelleplexes varying in molar ratio between the copolymer’s amine groups and the DNA’s phosphate groups (N/P) were obtained (N/P = 1:1, 5:1, 7.5:1, 10:1, and 20:1).

### 3.9. Ethidium Bromide Displacement Assay

Aqueous copolymer micellar dispersions (0.5 mL) prepared at different concentrations were added to equal volumes of DNA/EtBr complex solutions containing 10 μg DNA and 3.0 μg EtBr. Thus, micelleplexes with various N/P ratios (from 0.5:1 to 20:1) were formed and left to equilibrate for three hours. The emission intensity was measured at 604 nm (exited at 530 nm). The relative fluorescence intensity of the polyplexes was expressed as a percentage of that of the DNA/EtBr solution. The measurements were performed in triplicate.

### 3.10. MTT Test

The MTT assay was performed on a human carcinoma hepatocyte (HepG2) cell line. The cells were seeded at an initial concentration of 1 × 10^4^ cells mL^−1^ 24 h prior to treatment. The treatment with free quercetin, empty micelles, quercetin-loaded micelles, and quercetin-loaded micelleplexes with DNA was performed in DMEM cell medium with 10% fetal bovine serum and penicillin/streptomycin (Sigma–Aldrich/Merck, Darmstadt, Germany) for 24 h. Untreated cells were used as a control. After treatment, cells were incubated with 0.05 mg mL^−1^ MTT reagent (Sigma–Aldrich/Merck). The formazan crystals, that appeared as a result of intracellular reduction of MTT reagent by dehydrogenase enzyme activities, were dissolved with DMSO [50]. The absorbance of the samples was measured at a wavelength of 562 nm. Metabolic activity was calculated as the percentage of absorbance of the sample relative to the absorbance of the control.

### 3.11. Characterization Methods

^1^H NMR analyses were performed on a Bruker AV NEO 600 MHz (Bruker, Billerica, MA, USA) instrument with CDCl_3_ as a solvent. UV–Vis spectra were recorded on a DU 800 Beckman Coulter spectrometer (Beckman Coulter, Inc., Brea, CA, USA). The fluorescence measurements were conducted on an Agilent Cary Eclipse Fluorescence spectrophotometer (Santa Clara, CA, USA). Infrared spectra were obtained from an IRAffinity-1 Shimadzu Fourier Transform Infrared (FTIR) spectrophotometer (Kyoto, Japan) equipped with a MIRacle attenuated total reflectance attachment. The molar mass distributions of the (co)polymers were determined using gel permeation chromatography (GPC) using a Shimadzu Nexera XR HPLC chromatograph (Kyoto, Japan) equipped with a quaternary pump, degasser, automatic injector, column heater, UV/Vis (SPD-20A) detector, differential refractive index (RID-20A) detector, 10 μm PL gel mixed-B, and 5 μm PL gel 500 Å and 50 Å columns. The mobile phase was THF (+2 wt.% triethylamine) with a flow rate of 1.0 mL min^−1^. The system was calibrated with polystyrene narrow molar mass standards. Transmission electron microscope (TEM) images were acquired on an HRTEM JEOL JEM-2100 (200 kV) instrument (JEOL, Peabody, MA, USA) equipped with CCD camera GATAN Orius 832 SC1000 (Pleasanton, CA, USA). A GATAN Microscopy Suite 3.4 Software was used to process the images and to observe the morphology and particle size of the various types of nanoaggregates. The average diameters and size distribution of the prepared micelles and micellplexes were determined using dynamic light scattering (DLS) on a NanoBrook Plus PALS instrument (Brookhaven Instruments, New York, NY, USA), equipped with a 35-mW solid-state laser operating at λ = 660 nm at a scattering angle of 90° and using Particle Solutions (v.3.6.0.7136) software. The average hydrodynamic diameters (*d_H_*) of the nanoparticles were obtained by applying the Stokes–Einstein equation:

*d_H_* = *kT*/(*3πηD*) (3)

(*k*—Boltzmann’s constant, *T*—temperature (K), *η*—viscosity, *D*—diffusion coefficient).

The phase analysis light scattering was utilized to determine the electrophoretic mobility of the surface-charged micelles’ and micelleplexes’ dispersions. Thus, the particles’ ζ-potentials were derived by applying the Smoluchowski equation:

*ζ* = *4πημ*/*ε* (4)

(*ζ*—zeta potential (mV), *η*—viscosity; *μ*—electrophoretic mobility, *ε*—solvent’s dielectric constant).

The size and size distribution measurements were triplicated per run and were averaged from three independent runs. The zeta potential measurements were also triplicated per run and averaged from twenty runs.

## 4. Conclusions

An amphiphilic multifunctional block copolymer bearing pendant alkyne groups was used as a modular platform for the attachment of a desired number of preformed end-functional poly(2-(dimethylamino)ethyl methacrylate) (PDMAEMA) side chains. The “grafting to” approach was realized by applying an azide–alkyne “click” reaction. The obtained novel amphiphilic copolymer with block and graft architecture was characterized and used to form cationic micelles through self-association in aqueous media. The micelles were efficiently loaded with the natural hydrophobic drug quercetin. The micelles before and after drug-loading were physico-chemically characterized. They were further evaluated as potential carriers of both quercetin and DNA. Thus, nanosized micelleplexes with a narrow size distribution were successfully obtained through electrostatic interactions between the drug-loaded cationic micelles and DNA. The micelleplexes exhibited high colloidal stability. The performed initial in vitro biological evaluations indicate that the novel micelleplexes have a potential for further evaluation as safe nanocarriers for hydrophobic drug and DNA codelivery.

## Figures and Tables

**Table 1:** Characteristics of the polymer precursors and the cationic amphiphilic graft copolymer.

	Precursors				Cationic Amphiphilic Graft Copolymer				
Code	t-*DP*_n_ ^a^	*DP*_n_ ^b^	*M*_n_ ^b^ (g mol^−1^)	*Ð*_M_ ^c^	Code	t-*DG*_n_ ^d^	*DG*_n_ ^b^	*M*_n_ ^b^ (g mol^−1^)	*Ð*_M_ ^c^
MPEG-*b*-PC	-	-	9800	1.24	MPEG-*b*-(PC-*g*-PDMAEMA)	14	12	85,400	1.22
PDMAEMA-N_3_	30	40	6300	1.27					

^a^ Target degree of polymerization, t-*DP*_n_ = [DMAEMA]_0_/[Br]_0_, where [Br]_0_ is the active center concentration. ^b^ Number-average molar mass (*M*_n_), degree of polymerization (*DP*_n_), and degree of grafting (*DG*_n_), as determined through ^1^H NMR analyses. ^c^ Molar mass dispersity (*Ð*_M_), as determined through GPC analyses. ^d^ Target degree of grafting, t-*DG*_n_ = [Alkyne groups]_0_/[PDMAEMA-N_3_]_0_.

**Table 2:** Characteristics of the different MPEG-*b*-(PC-*g*-PDMAEMA)-based nanoaggregates.

Nanoaggregates (Code)	CMC ^a^ (mg mL^−1^)	d ^b^ (nm)	PdI ^b^	ζ ^b^ (mV)	DLE ^c^ (%)	DLC ^c^ (%)
Empty Micelles (M)	0.032	157.91 ± 2.42	0.221	20.08 ± 3.73	-	-
Drug-Loaded Micelles (M/Que)	-	126.88 ± 2.26	0.312	21.42 ± 4.10	93	8.5
Micelleplexes (MP 10:1)	-	91.80 ± 1.49	0.173	21.64 ± 1.65	-	-

^a^ Critical micelle concentration determined spectroscopically. ^b^ Average micelle diameters (d), size distributions (PdI), and zeta potentials (ζ) obtained from DLS measurements. ^c^ Drug-loading efficiency (DLE) and drug-loading capacity (DLC) are determined spectroscopically.

## Data Availability

Data are contained within the article and Supplementary Materials.

## Supplementary Materials

The following supporting information can be downloaded at: https://www.mdpi.com/article/10.3390/molecules29112540/s1, Figure S1: GPC overlay of the functional homopolymer PDMAEMA-N_3_; the backbone block copolymer MPEG-*b*-PC and the graft copolymer MPEG-*b*-(PC-*g*-PDMAEMA); Figure S2: FTIR spectra of (a) azide end-functional polymer (PDMAEMA) and (b) cationic amphiphilic graft copolymer MPEG-*b*-(PC-*g*-PDMAEMA); Figure S3: Size distributions (a) and zeta potentials (b) obtained from dynamic light scattering analysis of aqueous dispersions of the micelleplexes prepared at different N/P ratios: 1:1 (MP 1:1, d = 143.89 ± 0.86 nm, PdI: 0.148, ζ = −19.28 ± 2.27 mV), 2.5:1 (MP 2.5:1, d = 361.01 ± 5.92 nm, PdI: 0.194, ζ = 20.05 ± 1.88 mV), 5:1 (MP 5:1, d = 351.69 ± 6.20 nm, PdI: 0.187, ζ = 19.20 ± 4.06 mV), 7.5:1 (MP 7.5:1, d = 112.83 ± 0.98 nm, PdI: 0.210, ζ = 23.79 ± 2.10 mV), 10:1 (MP 10:1, d = 91.80 ± 1.49 nm, PdI: 0.173, ζ = 21.64 ± 1.65 mV), 20:1 (MP 20:1, d = 78.75 ± 0.29 nm, PdI: 0.172, ζ = 23.23 ± 1.89 mV); Figure S4: Size distribution curves obtained using DLS of aqueous micelleplex dispersion prepared at an N/P ratio of 10:1 after 60 min of incubation in an acidic buffer at pH 5.6.
