# Supplementary material for: Poly(2-(dimethylamino)ethyl methacrylate)-Grafted Amphiphilic Block Copolymer Micelles Co-Loaded with Quercetin and DNA
Source: Molecules. 2024 May 28;29(11):2540. doi: 10.3390/molecules29112540 (PMC11173910; doi:10.3390/molecules29112540)
Supplement: Supplementary file 1 [file molecules-29-02540-s001.zip › molecules-3011628-supplementary.pdf]

Supplementary Materials

Poly(2-(Dimethylamino)ethyl Methacrylate)-Grafted Amphiphilic Block Copolymer Micelles Co-Loaded with Quercetin and DNA

Radostina Kalinova [1,*], Pavel Videv[2], Svetla Petrova[2], Jordan Doumanov[2] and Ivaylo Dimitrov [1,*]

[1] *Institute of Polymers, Bulgarian Academy of Sciences, Akad. G. Bonchev St., bl. 103-A, 1113 Sofia, Bulgaria;*

[2] *Faculty of Biology, Sofia University "St. Kliment Ohridski", 8 Dragan Tzankov Blvd., 1164 Sofia, Bulgaria*

*Correspondence: kalinova@polymer.bas.bg; dimitrov@polymer.bas.bg

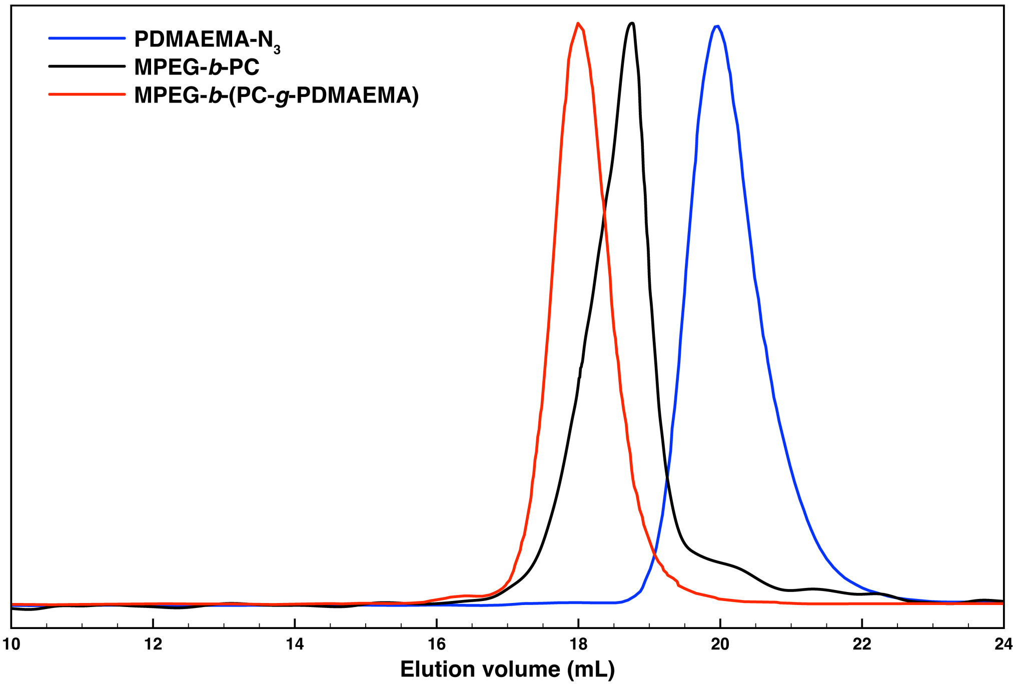

Figure S1. GPC overlay of the functional homopolymer PDMAEMA-N_3; the backbone block copolymer MPEG-*b*-PC and the graft copolymer MPEG-*b*-(PC-*g*-PDMAEMA).

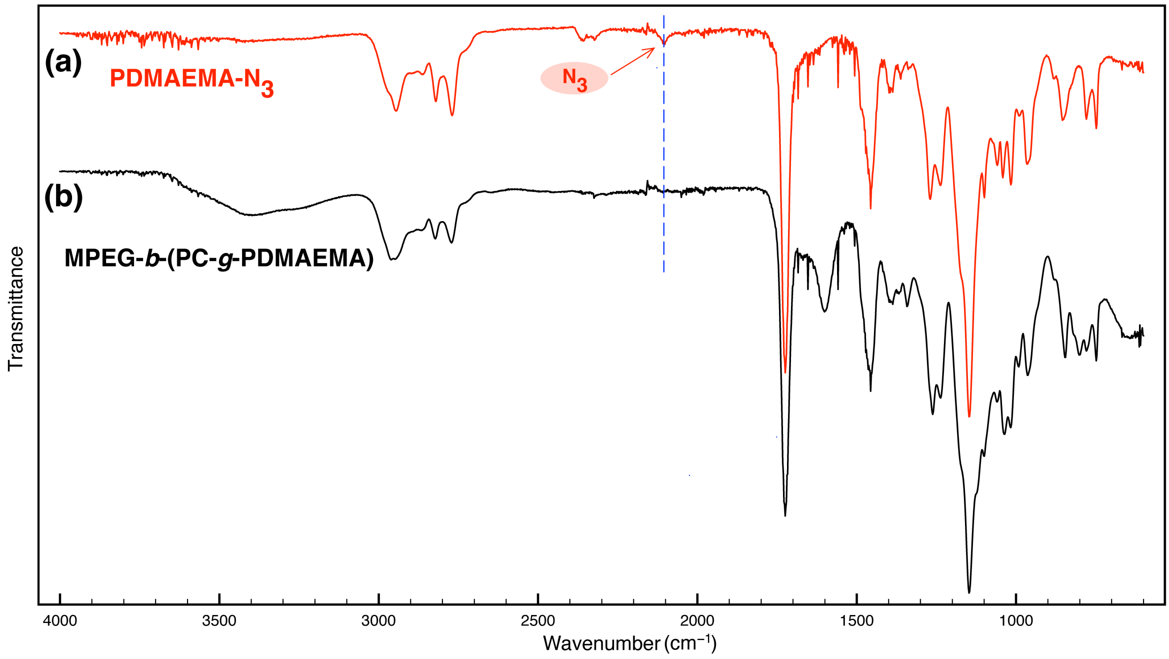

Figure S2. FTIR spectra of: (**a**) azide end-functional polymer (PDMAEMA-N_3); and (**b**) cationic amphiphilic graft copolymer MPEG-*b*-(PC-*g*-PDMAEMA).

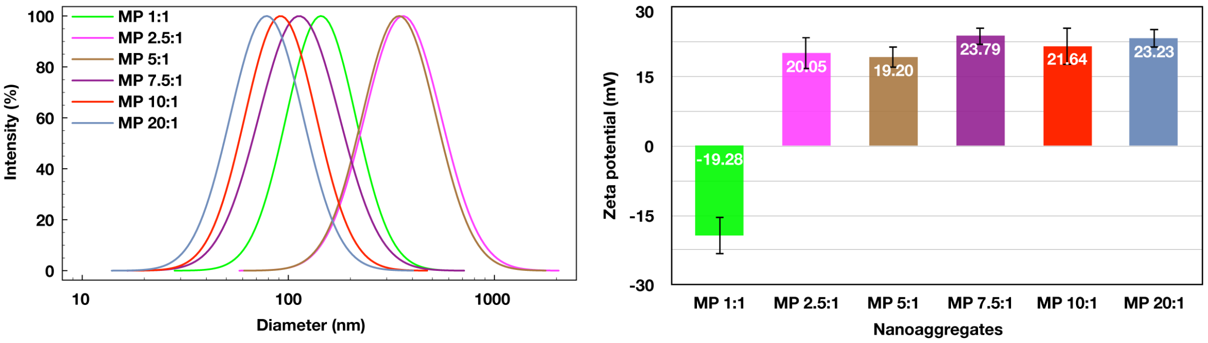

(a) **(b)**

Figure S3. Size distributions (**a**), and zeta potentials (**b**) obtained from dynamic light scattering analysis of aqueous dispersions of the micelleplexes prepared at different N/P ratios: 1:1 (MP 1:1, d = 143.89 ± 0.86 nm, PdI: 0.148, ζ = - 19.28 ± 2.27 mV), 2.5:1 (MP 2.5:1, d = 361.01 ± 5.92 nm, PdI: 0.194, ζ = 20.05 ± 1.88 mV), 5:1 (MP 5:1, d = 351.69 ± 6.20 nm, PdI: 0.187, ζ = 19.20 ± 4.06 mV), 7.5:1 (MP 7.5:1, d = 112.83 ± 0.98 nm, PdI: 0.210, ζ = 23.79 ± 2.10 mV), 10:1 (MP 10:1, d = 91.80 ± 1.49 nm, PdI: 0.173, ζ = 21.64 ± 1.65 mV), 20:1 (MP 20:1, d = 78.75 ± 0.29 nm, PdI: 0.172, ζ = 23.23 ± 1.89 mV).

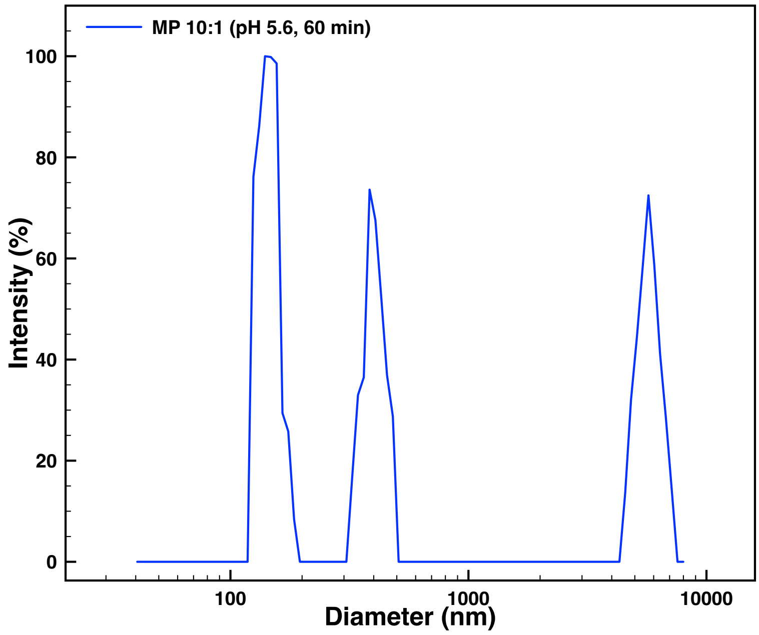

Figure S4. Size distribution curves obtained using DLS of aqueous micelleplex dispersion prepared at an N/P ratio of 10:1 (MP 10:1) after 60 min of incubation in an acidic buffer at pH 5.6.
